# Dramatically Enhanced Spin Dynamo with Plasmonic Diabolo Cavity

**DOI:** 10.1038/s41598-017-05634-0

**Published:** 2017-07-13

**Authors:** Peng Gou, Jie Qian, Fuchun Xi, Yuexin Zou, Jun Cao, Haochi Yu, Ziyi Zhao, Le Yang, Jie Xu, Hengliang Wang, Lijian Zhang, Zhenghua An

**Affiliations:** 1 State Key Laboratory of Surface Physics and Key Laboratory of Micro and Nano Photonic Structures (Ministry of Education), Department of Physics, Fudan University, Shanghai, 200433 China; 2 Collaborative Innovation Center of Advanced Microstructures, Fudan University, Shanghai, 200433 China

## Abstract

The applications of spin dynamos, which could potentially power complex nanoscopic devices, have so far been limited owing to their extremely low energy conversion efficiencies. Here, we present a unique plasmonic diabolo cavity (PDC) that dramatically improves the spin rectification signal (enhancement of more than three orders of magnitude) under microwave excitation; further, it enables an energy conversion efficiency of up to ~0.69 mV/mW, compared with ~0.27 μV/mW without a PDC. This remarkable improvement arises from the simultaneous enhancement of the microwave electric field (~13-fold) and the magnetic field (~195-fold), which cooperate in the spin precession process generates photovoltage (PV) efficiently under ferromagnetic resonance (FMR) conditions. The interplay of the microwave electromagnetic resonance and the ferromagnetic resonance originates from a hybridized mode based on the plasmonic resonance of the diabolo structure and Fabry-Perot-like modes in the PDC. Our work sheds light on how more efficient spin dynamo devices for practical applications could be realized and paves the way for future studies utilizing both artificial and natural magnetism for applications in many disciplines, such as for the design of future efficient wireless energy conversion devices, high frequent resonant spintronic devices, and magnonic metamaterials.

## Introduction

In 2007, Y. S. Gui, *et al*.^1^ first proposed and demonstrated the spin dynamo, first proposed and demonstrated the spin dynamo, is constructed that provides a new and interesting way to generate direct current via spin precessions to locally power nanoscopic devices and for future applications such as wireless energy conversion. Compared with the spin-driven currents in semiconductors^2^, spin dynamos are based on ferromagnetic materials^1^ or spin-torque diodes^3, 4^, which feature a much higher current/power ratio coupled with a much smaller internal resistance. However, the reported works are limited to sophisticated waveguide couplings (and therefore to wires), such as coplanar waveguides (CPWs)^5, 6^, microstrip lines^7, 8^, and bias Tees^3, 9–12^, to in-couple radio-frequency or microwave electromagnetic waves. Free space direct illumination has rarely been studied, despite its excellent suitability for wireless energy conversion. One main reason may be that the wireless conversion efficiency at present is extremely low to allow the spin dynamo to generate any discernible power.

In past decades, metamaterials or artificial resonant structures have emerged as an agile and promising way to manipulate electromagnetic fields at a deep subwavelength scale, leading to enhanced light-matter and light-spin interactions. For instance, a variety of intriguing new phenomena have been observed in plasmon-spin hybrid systems, such as the large enhancement of Faraday rotation via plasmonics^13^, plasmonics enhanced magneto-optical effects^14, 15^, and magneto-plasmonics^16–19^. Furthermore, T. Grosjean *et al*.^20^ have theoretically predicted a diabolo resonant antenna that should exhibit a large magnetic field enhancement reaching as high as 2700-fold. Metamaterials therefore offer an appealing solution to boost the coupling between electromagnetic waves and spins and hence an enhanced spin dynamo can be expected when exploiting them for this application^21^. For a spin dynamo based on spin rectification under ferromagnetic resonance (FMR) conditions^22^, simultaneous enhancements of both the electric and magnetic fields as well as the tunability of their mutual phase are anticipated. This is, however, nontrivial since electric and magnetic field enhancements from a pure plasmonic resonance typically occur at spatially different locations with a stubborn 90° phase deviation, as suggested based on the viewpoint of the equivalent LC resonance.

In this work, we combined a modified diabolo antenna (MDA) with a photonic structure and utilized the hybrid resonance to improve the spin dynamo performance. We demonstrate that the spin dynamo rectification signal can be improved by more than three orders of magnitude and that an energy conversion efficiency of up to ~0.69 mV/mW can be achieved thanks to the simultaneous enhancement of the microwave electric field (~13-fold) and the magnetic field (~195-fold) with a relative phase distinctive from 90°. Our work provides an innovative way to optimize spin dynamo performance and holds potential for general applications in the form of wireless high frequent spintronic devices such as magnetic tunnel junctions^15, 23, 24^, spin-torque diodes^3, 25^, spin pumping^26, 27^, and spintronic microwave sensors^28, 29^.

## Results

**Figure 1:**
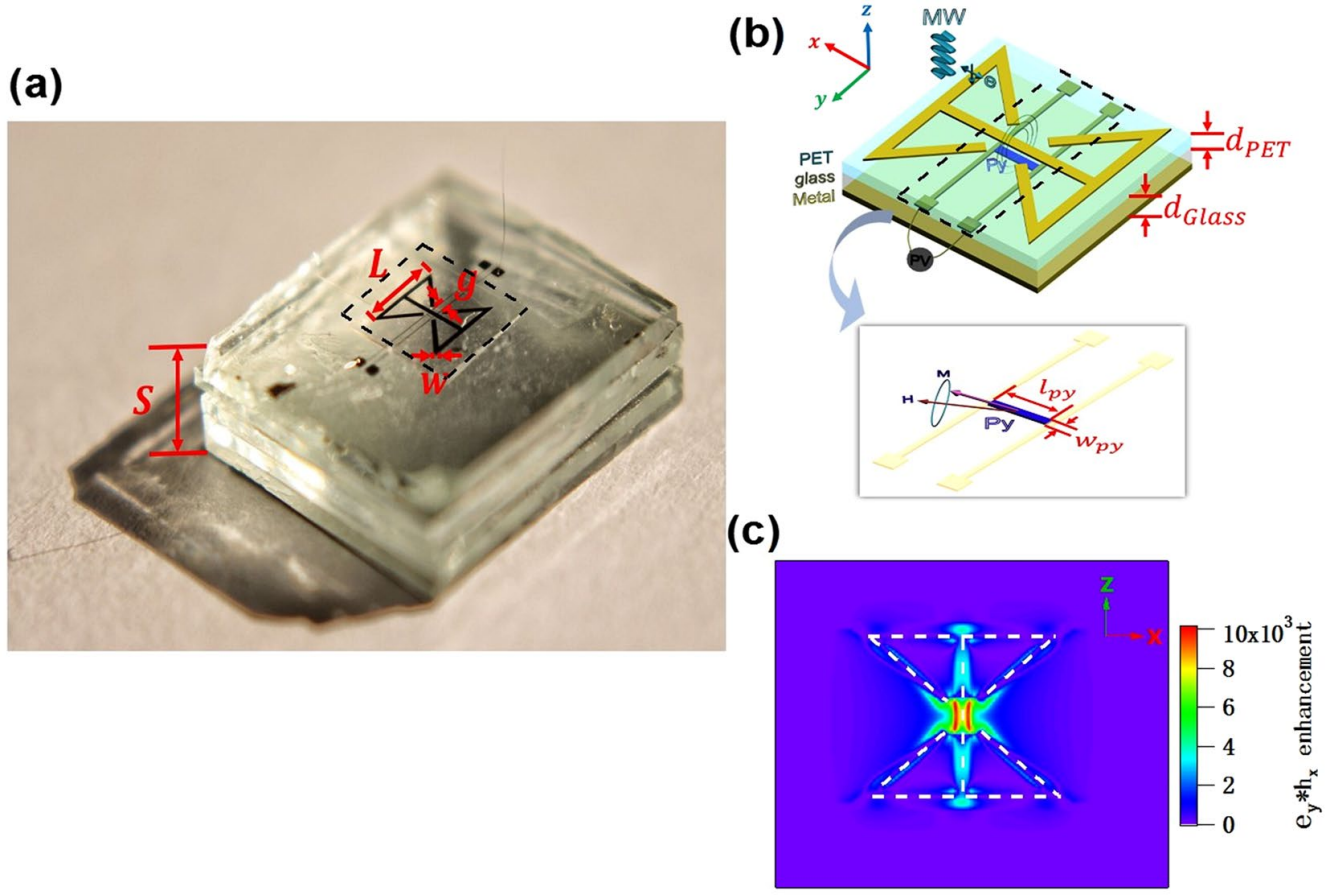
Schematic structure of the plasmonic diabolo cavity (PDC) and the simulation results. (**a**) Actual sample in our experiment, where the thickness of the cavity, *S*, was tunable. Typical dimension of MDA: *L* = 3 mm, *w* = 0.2 mm, and *g* = 0.5 mm. (**b**) Schematic diagram of the PDC, which contains three parts: the MDA structure on the PET, the spin dynamo device on the glass substrate, and the flat Al foil. Insert is the magnified image of spin dynamo, with a dimension: *l*_*py*_ = 600 *μm* and *w*_*py*_ = 20 *μm*. Panel (c) shows the enhancement of the product of the electric and magnetic field (*e*_z *_ *h*_*x*_) on the *x–z* plane.

Figure 1(a) shows the real sample and 1(b) shows a schematic drawing of the PDC’s designed metal/insulator/metal (MIM) sandwich structure; its top consists of a MDA (with dimensions of *L* × *L* mm^2^ in the *x–z* plane with a copper strip of width *w* and two pairs of copper strips (each with a gap of width *g*) evaporated onto a polyethylene terephthalate (PET) substrate. The layer sandwiched in the middle was glass (with dielectric constant ε = 6.8), while the bottom mirror layer was a flat pieces of Al foil (see Fig. 1(b)). The spin dynamo device (insert of Fig. 1(b)) was located 60 μm below the MDA. The MDA structure provides plasmon resonance with both localized e- and h-fields around the centre while the MIM tri-layer structure offers Fabry-Perot-like photonic resonance. The resulting hybridized mode functions to enhance both the electric and the magnetic field.

As the spin dynamo requires both electric and magnetic field enhancements, Fig. 1(c) shows the enhancement of the product between the electric field (*z* direction) and the magnetic field (*x* direction) at the monitor in the *x–z* plane calculated using a finite-difference time-domain (FDTD) simulation method. We can see that *e*_z*_ *h*_*x*_ is maximum in the centre region below the centre of the MDA. The ferromagnetic microstrip sample of Permalloy Ni_80_Fe_20_ (Py) for the spin dynamo is placed within this centre region (as shown in Fig. 1(b)) with the spin-rectifying photovoltage (PV) being measured via two parallel electrodes (Fig. 1(a) and (b)).

### Dramatically enhanced PV of spin dynamo

**Figure 2:**
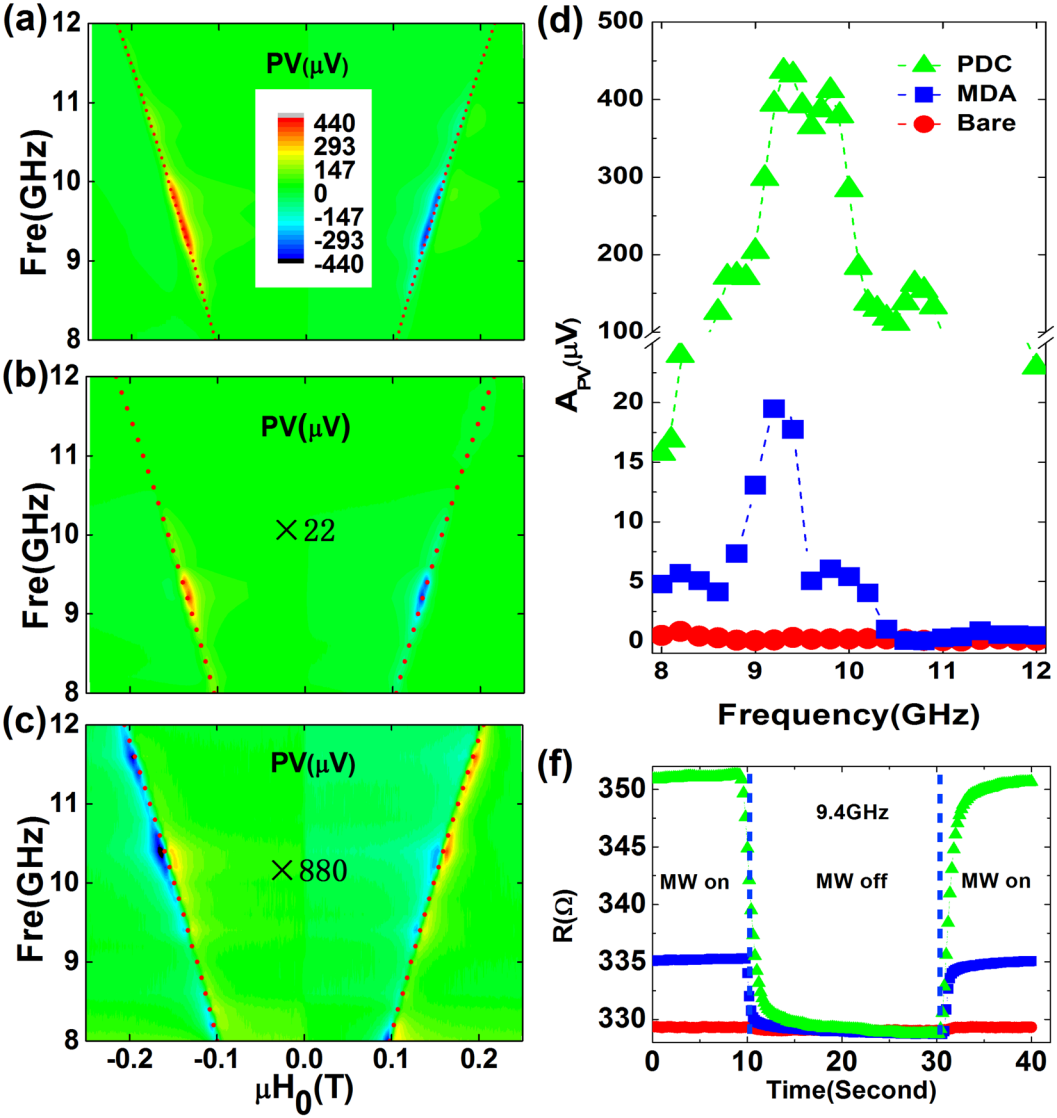
Experimental results. Panels (a)–(c) successively show the two-dimensional PV spectrum as a function of the external magnetic field and microwave frequency for three configurations: PDC, MDA, and bare. The red dashed lines indicate the calculated FMR frequency using Kittel’s formula ${\rm{\omega }}={\rm{\gamma }}\sqrt{|{H}_{0}|(|{H}_{0}+{M}_{0}|)}$. (**d**) The amplitude of microwave photovoltage (*A*_*PV*_) as a function of frequency. (**e**) The change of the sample’s resistance under microwave (9.4 GHz) irradiation with a period of 42 s (width 21 s) at 23 dBm with different configurations (PDC: green solid triangles; MDA: blue solid squares; and bare: red solid circles).

We began our experiment by applying a DC magnetic field with an angle of *θ* = 135° (the angle at which the largest PV is typically obtained); Fig. 2(a–c) shows the results for configurations with a PDC with a 3-mm-thick cavity, a MDA without the flat Al foil at the bottom, and a bare structure (without an MDA and without Al foil) with only a spin dynamo device, respectively. From the typical PV spectra, we can see that the normal FMR of these three conditions consistently follows Kittel’s formula ${\rm{\omega }}={\rm{\gamma }}\sqrt{|{H}_{0}|(|{H}_{0}+{M}_{0}|)}$ with $\frac{\gamma }{2\pi {\mu }_{0}}=21.5\pm 0.1GHz/T$ and a saturation magnetization *μ*_0_ *M*_0_ = 1.21 ± 0.02*T* as shown in the two-dimension spectrum (red dashed lines in Fig. 2(a)–(c)), which can be attributed to the intrinsic properties of the magnetic material (Py).

The spin dynamo PV induced by the spin rectification effect in the three configurations (as shown in Fig. 2(d)) near the FMR condition, shows a relatively small and non-resonant amplitude of ~0.17 μV (red solid circles) with the bare structure and ~17 μV (blue solid squares) with the MDA, resonant at a frequency of ~9.4 GHz. More remarkably, the PV with the PDC was as large as ~432 μV (at ~9.4 GHz, green solid triangles), constituting an enhancement factor of ~2541, which is much larger than in the pure plasmonic case with only a MDA (~100-fold enhancement). The conversion ratio (defined as the PV, *A*_*PV*_, divided by the microwave excitation power, *P*_*MW*_) achieved 0.69 mV/mW with the PDC—a record efficiency for wireless power conversion in spin dynamo.

To evaluate the respective contribution of the electric field *e*_*z*_ and the magnetic field *h*_*x*_, the bolometric effect^30^ was examined with different configurations but without an external DC magnetic field. As is pointed out in ref. 30, the resistance change (Δ*R*) of the Py strip caused by the bolometric effect under microwave irradiation satisfies ${\rm{\Delta }}R=({P}_{0}\tau /C)\partial R/\partial T$, where *P*_*0*_ is the absorbed microwave power, *τ* is the thermal energy relaxation time, *C* is the absolute heat capacity of the spin dynamo (i.e. of the Py stripe). Meanwhile, the electric field correlates with the resistance change:$\,{e}_{z}\propto {j}_{z}\propto \sqrt{{P}_{0}}\propto \sqrt{{\rm{\Delta }}R}$. Therefore, we can calculate the enhancement of *e*_*z*_ by measuring Δ*R*. In our experiment, a lock-in amplifier with an applied sine current (3.13 kHz, 0.17 μA) was used to measure the resistance of the Py strip, which was pulsed with microwaves (9.4 GHz) for a period of 42 s. As shown in Fig. 2(e), the resistance change (Δ*R*) jumps from 0.18 Ω (red solid circles) for the bare structure up to 30.02 Ω (green solid triangles) for the PDC and up to 4.44 Ω (blue solid squares) for the MDA. These resistance changes lead to a ~13-fold electric field enhancement (ξ_e_) for *j*_*z*_ or *e*_*z*_, which is too small to explain the observed PV enhancement (~2541×). Consequently, the additional enhancement can be ascribed to the enhancement of the microwave magnetic field (ξ_h_), which is approximately evaluated to be ~195 = 2541/13 at the resonant frequency (~9.4 GHz). Compared with the case for the pure MDA structure, where ξ_e_ ≈ 5 and ξ_h_ ≈ 20 = 100/5 at resonant frequency, the PDC structure shows a larger enhancement of both the electric and the magnetic field.

### Line shape of FMR caused by relative phase in PDC

**Figure 3:**
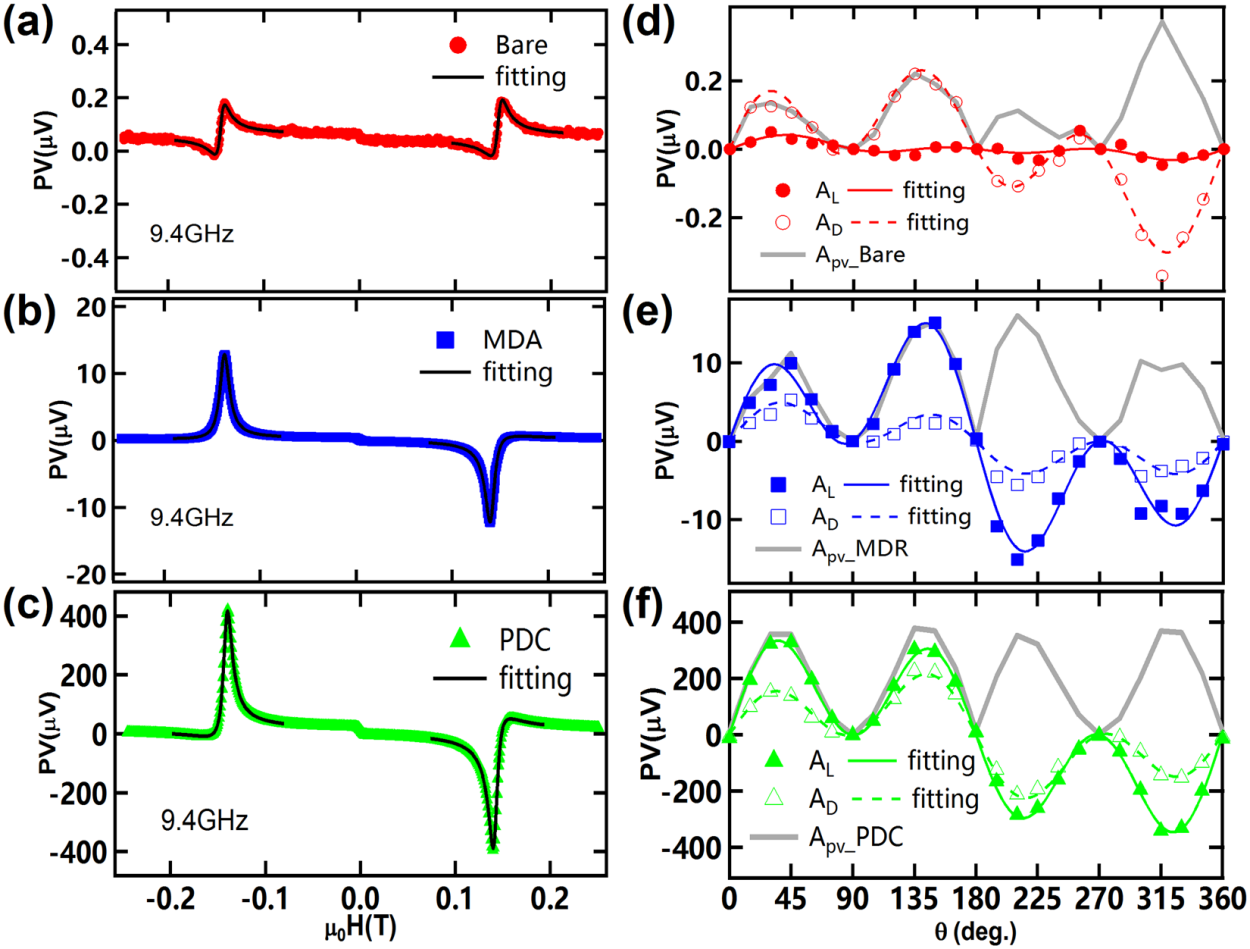
Line shapes of PV and Lorentzian and dispersive amplitudes for three structures. Panels (a)–(c) show the measured microwave PV line shapes for the PDC, MDA, and bare structures (at 9.4 GHz), respectively. Panels (d)–(f) show *A*_*L*_, *A*_*D*_ and *A*_*PV*_ with respect to *θ*.

To take account of the spectral line shape near the FMR, we then analysed the spin rectification effect more quantitatively^22^. Taking the time average <> of the electric field integrated along the *z* direction, we get $$PV=\frac{{\rm{\Delta }}R}{{M}_{0}}\langle Re(\begin{array}{c} \sim \\ j\end{array})\cdot Re(\begin{array}{c} \sim \\ m\end{array})\rangle ,$$ where Δ*R* is the resistance change caused by the anisotropic magnetoresistance (AMR) effect, *j*, is the microwave current in the Py strip induced by the microwave *e*-field, and *m* is the non-equilibrium magnetization driven by the microwave *h*-field. Figure 3(a–c) displays the DC voltage as a function of the DC magnetic *h*-field at 9.4 GHz. The line shape of the FMR can be fitted well (as shown by black lines) by the following equation consisting of a linear combination of dispersive and Lorentzian line shape components

1 $$PV={A}_{L}\frac{{\rm{\Delta }}{H}^{2}}{{(H-{H}_{0})}^{2}+{\rm{\Delta }}{H}^{2}}+{A}_{D}\frac{{\rm{\Delta }}H(H-{H}_{0})}{{(H-{H}_{0})}^{2}+{\rm{\Delta }}{H}^{2}}$$

where *A*_*L*_ and *A*_*D*_ are the amplitudes for the Lorentzian and dispersive components, respectively, Δ*H* is the line width, and *H*_*0*_ is the resonant magnetic field. We define the amplitude of the PV at the FMR to be ${A}_{PV}=\sqrt{{A}_{L}^{2}+{A}_{D}^{2}}$, as shown in Fig. 2(d).

From Fig. 3, we find that the line shape of the FMR is quite different at the angle of *θ* = 135° for the three structures: for the bare structure the FMR line shape is closer to the dispersive line shape, for the MDA structure it is closer to the Lorentzian line shape, while for the PDC structure it is somewhere between that of the dispersive and Lorentzian line shape. To understand the origin of the different line shapes, it should be noted that the spin rectification effect leads to different amplitudes of the Lorentzian (*A*_*L*_) and dispersive (*A*_*D*_) components depending on the relative phases between the microwave magnetic field *h* and the microwave current *j* (or electric field, *e*_*z*_); the microwave electric field (Φ_*x*_, Φ_*y*_, and Φ_*z*_, in the *x*, *y*, and *z* directions, respectively) and both *A*_*L*_ and *A*_*D*_ are represented in the following equations^31^:

2 $$\begin{array}{rcl}{A}_{L} & = & {\rm{\Delta }}R{j}_{z}\,{\sin }\,(2\theta )[-{A}_{xx}{h}_{x}\,{\sin }\,{({\rm{\Phi }}}_{x})\,{\cos }\,(\theta )+{A}_{xx}{h}_{z}\,{\sin }\,{({\rm{\Phi }}}_{z})\,{\sin }\,(\theta )\\  &  & -{A}_{xy}{h}_{y}\,{\cos }\,{({\rm{\Phi }}}_{y})]/2{M}_{0}\end{array}$$

3 $$\begin{array}{rcl}{A}_{D} & = & {\rm{\Delta }}R{j}_{z}\,{\sin }\,(2\theta )\,[{A}_{xx}{h}_{x}\,{\cos }\,{({\rm{\Phi }}}_{x})\,{\cos }\,(\theta )-{A}_{xx}{h}_{z}\,{\cos }\,{({\rm{\Phi }}}_{z})\,{\sin }\,(\theta )\\  &  & -{A}_{xy}{h}_{y}\,{\sin }\,{({\rm{\Phi }}}_{y})]/2{M}_{0}\end{array}$$

where Δ*R* and *θ* are the resistance change caused by the AMR effect and the angle between the *H* and Py stripe, respectively. As already mentioned, *j*_*z*_, is the microwave current along the Py strip and the pre-factors *A*_*xx*_, *A*_*xy*_, and *A*_*yy*_ are real numbers that are related to the Py properties. From Eqs (2) and (3) it can be seen that for the case where *Φ*_*x*_ = *Φ*_*y*_ = *Φ*_*z*_ = *0*, the dispersive component *A*_*D*_ dominates the line shape, leading to an antisymmetric shape, while for *Φ*_*x*_ = *Φ*_*y*_ = *Φ*_*z*_ = *π/2*, the Lorentzian component *A*_*L*_ dominants the line shape, leading to a symmetric shape^31^.

The *θ* dependent experiments (conducted by changing the orientation of *H* relative to the Py strip on *x–z* plane) show the variation of *A*_*L*_ and *A*_*D*_ (hollow/solid circles/squares/triangles, respectively, in Fig. 3(d)–(f)). We noted that *h*_*x*_ is the dominant component in our configuration (*h*_*x*_ ≫ *h*_*y*_ *, h*_*z*_), thus both of *A*_*L*_ and *A*_*D*_ are found to follow a *sin*(2*θ*)·cos(*θ*) dependence on the external DC magnetic field angle. In Fig. 3(d)–(f) it can be observed that the PV signal undergoes a transition from *A*_*D*_-dominance to *A*_*L*_-dominance after introducing the MDA, but the proportion of *A*_*D*_ increased with the PDC configuration. That is, the line shape transformed from a dispersive to a Lorentzian shape (as shown in Fig. 3(a,b)), while the line shape in the PDC configuration was a mix of both the dispersive and the Lorentzian shape (shown in Fig. 3(c)). Through curve fitting we can calculate the relative phase when using a MDA (Ф_*x*_ = −71.5°) or a PDC structure (Ф_*x*_ = −59.9°); these values differ greatly from when using a bare structure (−6.37°, Fig. 3(d)). These values agree reasonably well with the theoretical predictions that the relative phase when using a pure plasmonic MDA structure should be closer to −π/2 while it should be 0 for the bare structure (plane wave or photonic resonance case). The distinctive value of Ф_*x*_ = −59.9° for the PDC configuration, which diverges from both −π/2 and 0, suggests that the dramatically enhanced PV arises both from plasmonic and photonic resonances. Meanwhile, we can see that *A*_*PV*_ reach the maximum at $\theta =45^\circ ,135^\circ ,225^\circ ,315^\circ$ as shown in Fig. 3(d)–(f) (gray lines).

### Fabry-Perot-like photonic resonance of PDC

**Figure 4:**
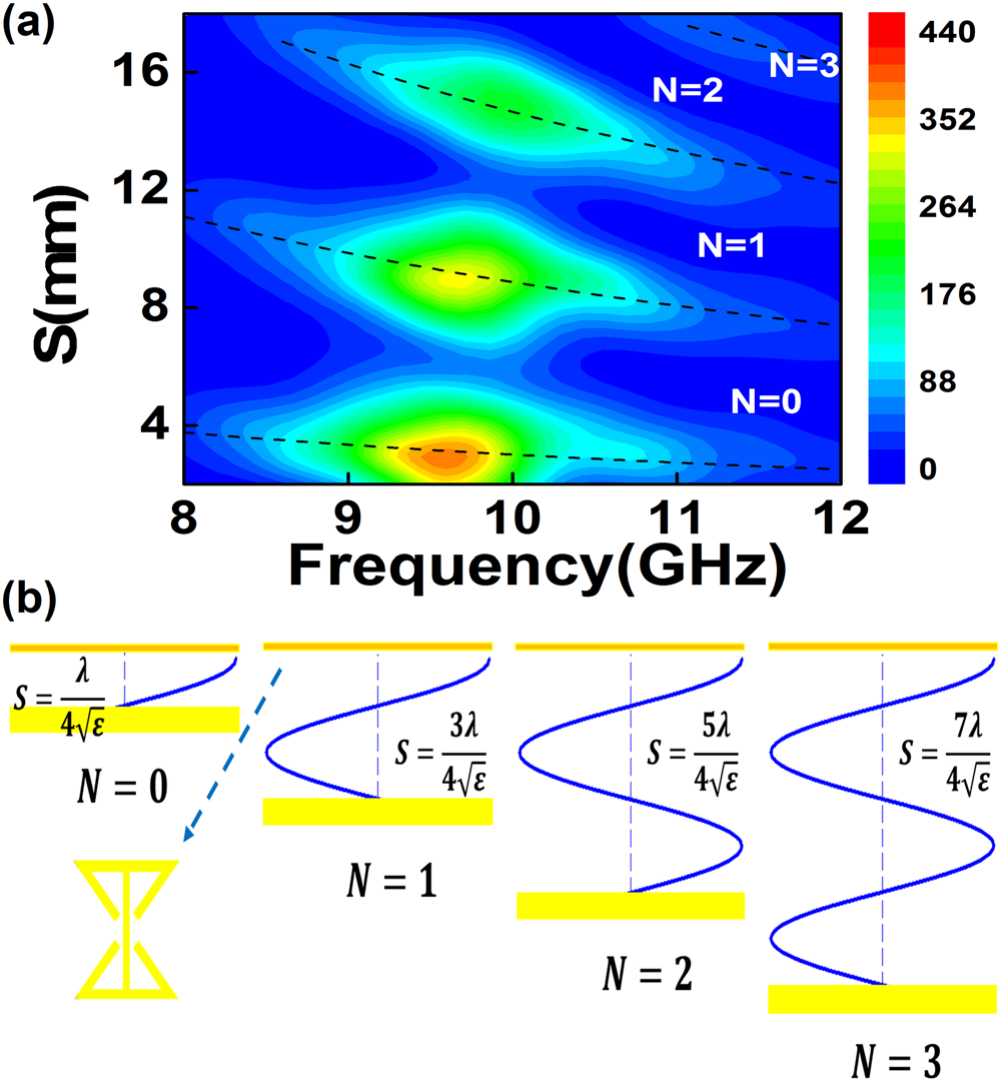
FP-like mode and visual theory. (**a**) Plot of the PV as a function of microwave frequency (8–12 GHz) and cavity thickness (2–18 mm). (**b**) The thickness (*S*) of the cavity corresponding to the orders (*N*) of the cavity mode in our photonic-like resonant mode.

To verify the contribution from the photonic-like resonant mode, we systematically varied the thickness *S* of the cavity and examined the enhancement of the PV signal. Figure 4(a) shows the two-dimensional plot of the PV spectrum as a function of the microwave frequency (8–12 GHz) and of the thickness of the cavity (2–18 mm). It is obvious that the enhanced PV band displays a systematic evolution as *S* increases. To determine the physical origins of these resonances, the dotted curves in Fig. 4(a) demonstrate the expected Fabry-Perot-like modes, which follow

4 $${\rm{S}}=(N+\frac{1}{2})\cdot \frac{c}{2\sqrt{\varepsilon }}\cdot \frac{1}{f}\,(N=0,1,2\cdots )$$

where *N* is the order of the cavity mode; *c* and *ε* are the velocity of light and the dielectric constant of glass, respectively; and *S* is the thickness of the PDC. Note that the electromagnetic field near the sample surface or the MDA should be close to the maxima associated with the spin-rectifying PV we detected (the hot sport in Fig. 4(a) shows the maximum PV); a straightforward physical model of this is demonstrated in Fig. 4(b), where the difference between the photonic-like resonant mode and the traditional Fabry-Perot mode^32^ is that a 1/2 item is added to accommodate the hybrid mode and where the thicknesses of the cavities for different orders are $\frac{\lambda }{4\sqrt{\varepsilon }},\frac{3\lambda }{4\sqrt{\varepsilon }},\frac{5\lambda }{4\sqrt{\varepsilon }}$ and $\frac{7\lambda }{4\sqrt{\varepsilon }}$, as shown in Fig. 4(b).

## Discussion

In summary, we proposed a novel PDC structure composed of a MDA and a flat metal layer, which has the ability to significantly enhance the spin dynamo rectification signal (by almost three orders of magnitude) and achieve a high-energy conversion efficiency ~0.69 mV/mW. We experimentally obtained an enhancement factor of ~2541× for PV, ~195× for the microwave magnetic field, and ~13× for the microwave electric field at the resonant frequency (9.4 GHz). Besides, the PDC structure also could modulate the relative phase of *e*- and *h*-field wildly via sophisticated design due to its hybrid mode, which originated from two resonant effect: plasmon resonance provided by the MDA structure, the relative phase close to π/2 corresponding a Lorentzian shape of FMR; the Fabry-Perot-like photonic resonance offered by the MIM tri-layer structure, quite different with the conventional Fabry-Perot cavity mode, and our theory explain the distinct phenomenon well.

Our work opens a door for future studies utilizing both artificial and natural magnetism, and further improvements can be considered in the following two aspects: Firstly, MIM structure could achieve perfect absorption^33^ of light, which provide a possibility to dramatically enhance the spin relevant effects because it would increase their energy conversion efficiency for the above-mentioned devices; then the plasmonic diabolo cavity structure could be developed into a perfect metamaterial absorber. Secondly, because the anisotropic magnetoresistance (AMR) effect of a single permalloy strip is not efficient ( < 1%) — therefore, much higher spintronic rectification effect such as giant magnetoresistance (GMR ~70%^34^), tunneling magnetoresistance (TMR ~600%^35^), or colossal magnetoresistance (CMR ~127000%^36^) can be adopted for future applications. The broad range of prospects for research in artificial and natural magnetism promises many exciting possibilities for the realization of efficient wireless energy conversion devices and wireless control devices in future.

## Methods

### Sample fabrication

In the experiment, standard optical lithography and Magnetron sputtering methods were used; a MDA copper structure with a thickness t_copper_ of 500 nm was fabricated on a 60-μm-thick PET substrate. It was then integrated into a ferromagnetic permalloy (Py or Ni_80_Fe_20_) microstrip sample (typically 600 μm × 20 μm × 40 nm) with gold electrodes (thickness t_gold_ of 200 nm) supported by a glass substrate. The bottom consisted of a flat metal (Al) layer to form a PDC device.

### Spin Rectification measurement setup

To measure the spin rectification photovoltage of Py, an external DC magnetic field was applied in the x–z plane with an angle *θ* with respect to the Py strip (*z* direction). A microwave generator (Agilent E8257D) whose amplitude was modulated by a square wave with a period of 0.12 ms, emitted an 8–12 GHz band electromagnetic wave through an honour antenna with its polarization along the z direction to normally illuminate the sample (i.e., it propagated along the y-direction). We detected the microwave SR PV generated in the Py strip by using a lock-in amplifier (Stanford SR830) triggered by the square wave. All the measurements were performed at room temperature.
